# Successful Treatment of Sternal Osteomyelitis and Mediastinitis Caused by Rhizopus Following Cardiac Surgery

**DOI:** 10.7759/cureus.62312

**Published:** 2024-06-13

**Authors:** Toot Chaw Tiew, Nur Ayub Md Ali, Muhammad Ishamuddin Ismail, Mohd Ramzisham Abdul Rahman, Chuan Hun Ding, Mohd Nizam Tzar

**Affiliations:** 1 Surgery, Faculty of Medicine, The National University of Malaysia, Kuala Lumpur, MYS; 2 Cardiothoracic Surgery, Faculty of Medicine, The National University of Malaysia, Kuala Lumpur, MYS; 3 Medical Microbiology and Immunology, Faculty of Medicine, The National University of Malaysia, Kuala Lumpur, MYS

**Keywords:** mucorales, zygomycosis, osteomyelitis, fungal infection, cardiac surgery, rhizopus, median sternotomy wound, mediastinal mucormycosis

## Abstract

Sternal osteomyelitis and mediastinitis are rare yet severe complications post-cardiac surgery, often associated with significant morbidity and mortality. Fungal etiologies, particularly those caused by *Rhizopus* spp., are infrequent but can lead to aggressive infections. Here, we present the case of a 68-year-old male who developed sternal osteomyelitis and mediastinitis caused by *Rhizopus *spp. two weeks following coronary artery bypass grafting surgery. Debridement and pectoralis flap reconstruction were performed following clinical identification and confirmation with microbiological examinations and a CT scan. Prompt recognition, aggressive surgical intervention, and targeted antifungal therapy were crucial for successful management. This case underscores the importance of considering fungal pathogens, such as *Rhizopus*, in the differential diagnosis of post-cardiac surgery infections, as well as aggressive treatment to improve outcomes for affected patients.

## Introduction

Mucormycosis is a potentially fatal invasive fungal infection caused by members of the Mucoraceae family, including *Lichtheimia*, *Mucor*, and *Rhizopus* species. Paltauf documented the first instance of this phenomenon in 1885 [1]. These pathogens are often found in decaying vegetated soils and mainly affect immunosuppressed populations, such as those with poorly controlled diabetes mellitus, prolonged steroid usage, hematological malignancies, iron overload states, and organ transplant recipients [2]. Mucormycosis can have various presentations based on its location in the body, such as rhino-cerebral, pulmonary, cutaneous, gastrointestinal, disseminated, and other unusual presentations (such as osteomyelitis, endocarditis, peritonitis, and pyelonephritis) [3]. However, it is extremely rare for it to occur as a sternal wound infection, which is a serious and debilitating complication of cardiac surgery. This can lead to delayed wound healing and progress to life-threatening complications such as sternal osteomyelitis and mediastinitis, which present significant challenges in patient management and outcomes. Here, we describe a unique case of postoperative sternal osteomyelitis and mediastinitis caused by *Rhizopus* following coronary artery bypass graft (CABG) surgery, which was treated with both surgical and medical management.

## Case presentation

**Figure 1:**
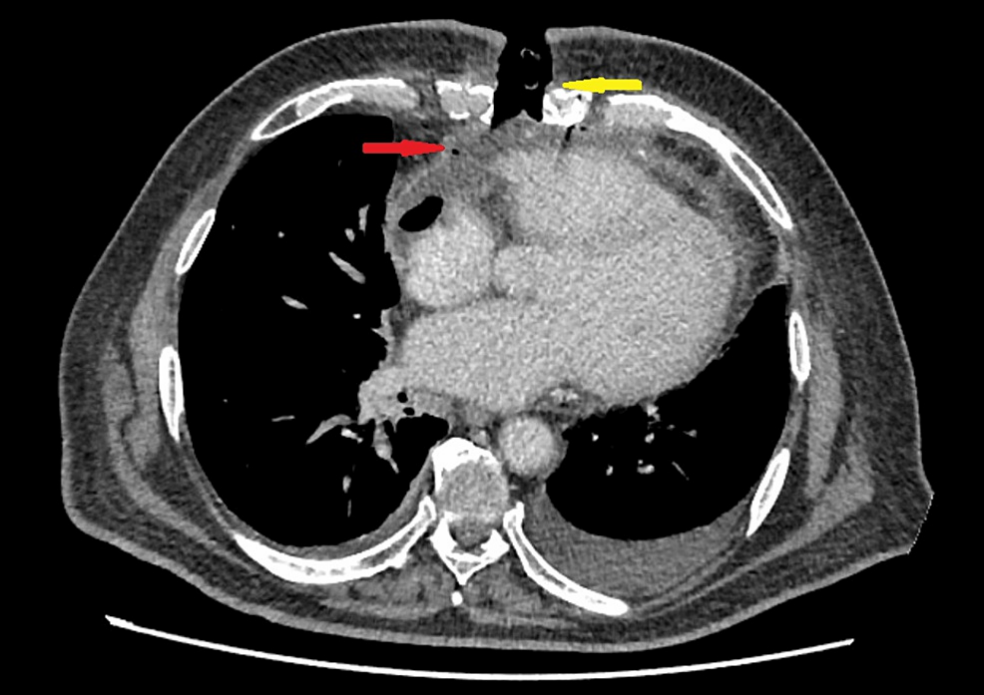
CT scan (axial cut) showing the midline sternotomy wound defect and sternal dehiscence (yellow arrow), with features of mediastinitis evidenced by retrosternal fluid and free air locules (red arrow).

**Figure 2:**
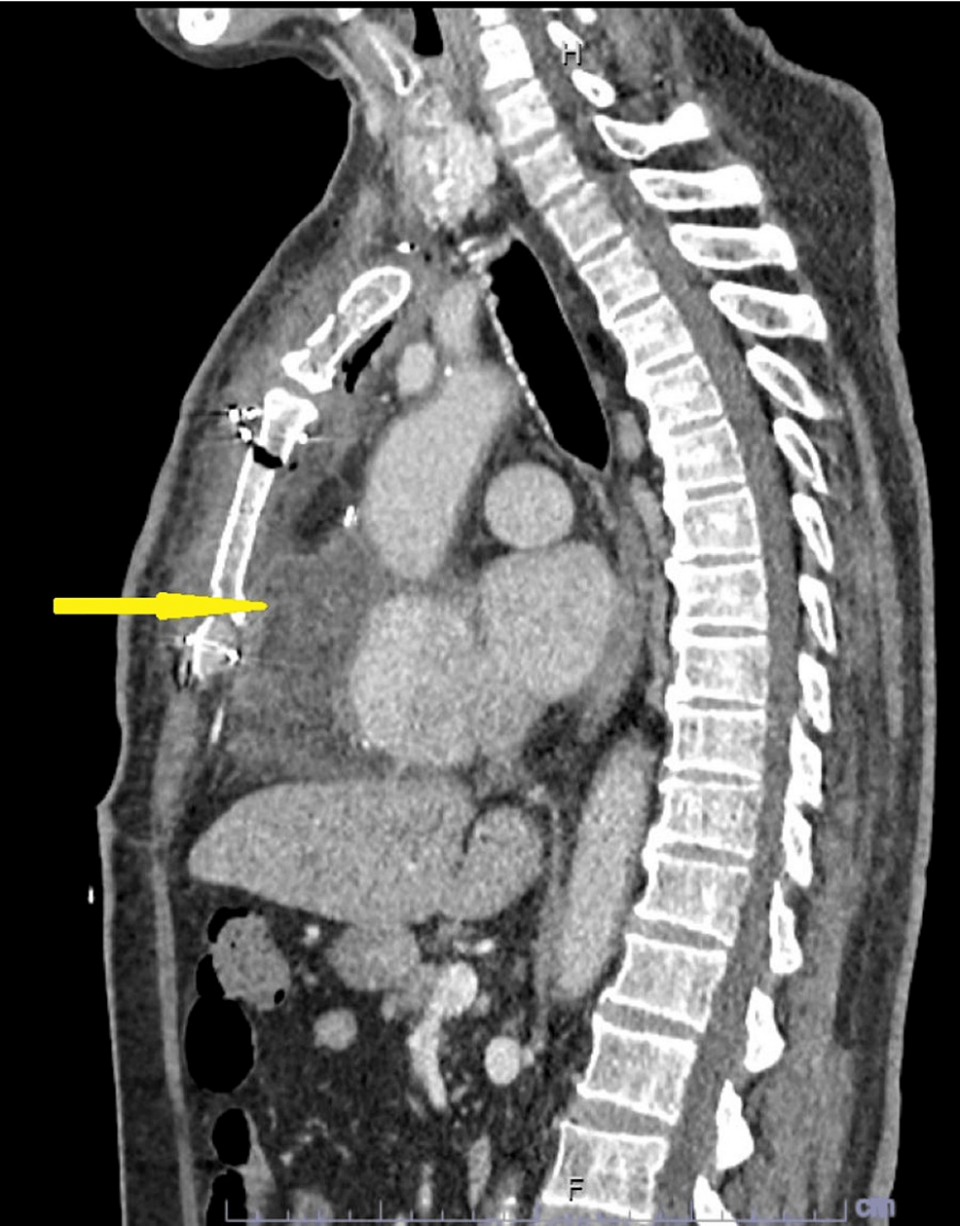
CT scan (coronal view) depicting anterior mediastinal collection (yellow arrow) with air locules and enhancing pericardium, suggestive of infected pericardial effusion.

A 68-year-old man with type 2 diabetes, hypertension, dyslipidemia, and three-vessel coronary artery disease underwent a CABG via the median sternotomy approach. His surgery and recovery went smoothly, and he was discharged six days after the surgery. However, on the 12th day after surgery, he experienced sternal pain, dyspnea during exertion, and purulent discharges from his sternal gaping wound due to severe coughing. The patient was not feverish, chilling, or showing any other systemic signs of infection. Chest X-ray was unremarkable. The patient’s purulent discharge was found to contain coagulase-negative staphylococci, initially regarded as skin flora, and the blood culture was sterile. He was treated with intravenous ceftazidime and started negative-pressure wound therapy (NPWT). A contrast-enhanced CT (CECT) scan of the chest showed a midline sternotomy wound defect and sternal dehiscence, indicating mediastinitis (Figures 1, 2).

**Figure 3:**
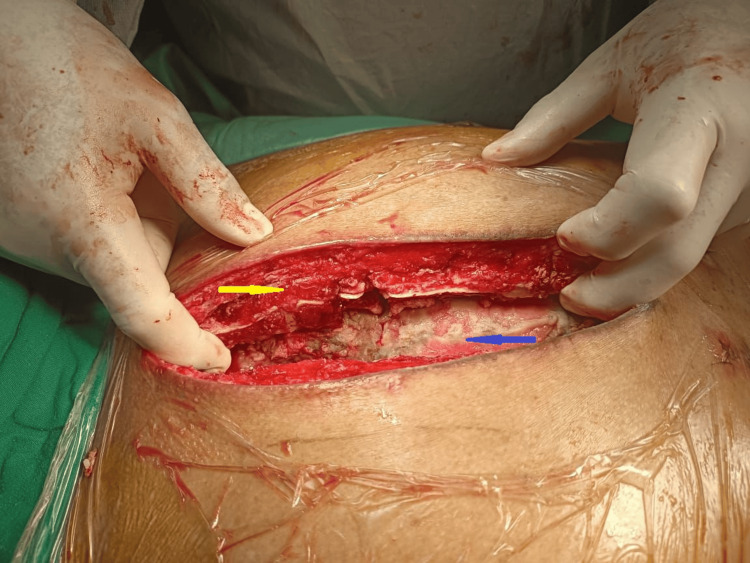
The appearance of sternal dehiscence, with bilateral marrow areas of the sternum extensively infiltrated with granulation tissue (yellow arrow) and sloughy anterior mediastinum (blue arrow) can be observed intraoperatively.

The patient was taken to the operating room to have six sternal wires removed, the wound debrided, and vacuum-assisted closure was applied to the sternotomy wound. Intraoperatively, sloughy tissue was noted throughout the anterior mediastinum, and the sternum had multiple shear fractures caused by the sternal wire. The intraoperative cultures taken during the operation came back negative. It was planned to reconstruct the sternum using the Robiscek procedure, which entails the placement of interlocking parasternal running wires on both sides and the inclusion of these wires in the usual transverse peristernal wires. During the second operation, however, this procedure was abandoned as the sternum was fragile, and bilateral marrow areas were extensively infiltrated with granulation tissue (Figure 3).

**Figure 4:**
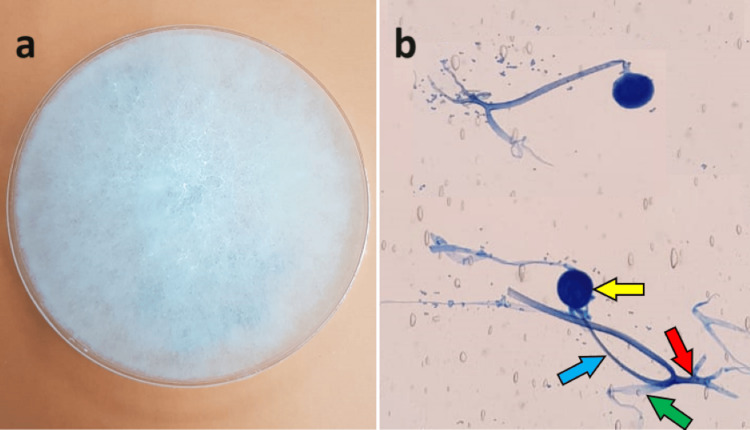
The fast-growing fungus covering an entire Sabouraud dextrose agar plate with a dense white cottony growth within the first week of culture (a). When examined microscopically (b), the fungus appears as an aseptate mold with stolons (green arrow) and rhizoids (red arrow). The sporangiophores (blue arrow) attached to globose sporangia (yellow arrow) arise from the nodes directly above the rhizoids.

Following extensive wound debridement, the decision was made to perform plastic surgery reconstruction and successfully close the open sternal wound using a pectoralis major muscle flap. The mediastinal area was irrigated with vancomycin powder and covered with gentamicin-impregnated collagen sheets (Collatamp®) before the sternal wound was closed, which may help localized control of the infection. An intraoperative sample of bone and tissue specimen grew *Staphylococcus haemolyticus* as well as a fungus identified as *Rhizopus* spp. (Figure 4).

After consultation with an infectious disease expert, the patient was administered an initial dose of vancomycin, followed by a maintenance dose intravenously. The patient also began oral isavuconazole at a daily dose of 200 mg after an initial loading dose. The medications were well tolerated, with no reported toxic effects. However, the patient experienced persistent fluid collection in the mediastinal space after surgery, necessitating pigtail drainage and daily aspirations. This resulted in a three-week hospitalization due to postoperative mediastinitis. During this time, consecutive CECT scans of the chest showed significant improvement in the anterior mediastinal collection and resolution of the mediastinitis. The patient experienced a positive recovery and was discharged home with a six-week continuation of antifungal treatment with isavuconazole.

## Discussion

Mucormycosis is an angioinvasive infection caused by aseptate fungi from the Mucorales order, class Zygomycetes. The most frequent culprits of mucormycosis confirmed through cultures are from the *Rhizopus* genus, resulting in potentially fatal infections in humans [4]. As a saprotrophic fungus, *Rhizopus* is a cosmopolitan fungus ubiquitous in soil, animal excrement, and decaying vegetation. *Rhizopus* spp. are typified by a mass of branching mycelia that consists of three types of aseptate hyphae: stolons, rhizoids, and sporangiophores, as shown in Figure 4. The sporangia at the tips of the sporangiophores are globose (rounded) and house numerous spores for asexual reproduction. *Rhizopus* colonies grow rapidly and fade from white to dark as spore production advances. They typically have a texture resembling cotton candy.

Traditional risk factors for mucormycosis include diabetes, hematological cancers, organ and bone marrow transplant recipients, treatment with deferoxamine, illicit intravenous drug use, and kidney failure [2]. Nonetheless, these conditions can also arise in individuals without any recognized risk factors. Patients without predisposition often manifest with skin and nasal sinus afflictions [3]. Heart complications in mucormycosis are rare and typically develop in severe infections in individuals with pre-existing conditions or those who have undergone heart surgery [5]. In our case, the development of mucormycosis following sternotomy may have been attributed to the patient’s history of diabetes. Similar cases have been reported in diabetic patients following coronary artery bypass surgery and mitral valve replacement. These patients rapidly developed invasive mucormycosis affecting the sternum and mediastinum, leading to their ultimate demise [6,7]. Our patient had mucormycotic sternal osteomyelitis and mediastinitis caused by *Rhizopus* following cardiac surgery, which responded well to surgical debridement and antifungal treatment. It is our understanding that this is the first reported case of *Rhizopus* mucormycosis associated with a median sternotomy wound in Malaysia.

It has been reported that the incidence of deep sternal wound infection after cardiac surgery is around 2%, with a mortality rate of 55% [8]. It is rare to have a deep sternal wound infected with fungi, especially *Rhizopus* species, whereas invasive candidiasis and aspergillosis represent the leading cause of invasive mold infections [9]. It is often difficult to diagnose clinically and requires a high degree of clinical suspicion. The treatment of mucormycosis requires a multidisciplinary approach, which involves a combination of thorough surgical debridement of affected tissues, correction of risk factors, and systemic antifungal therapy. Several surgical strategies may be effective in treating fungal osteomyelitis and mediastinitis [8,10]. The outcomes of the different surgical approaches, however, did not differ significantly, although Abu-Omar et al. reported more promising results with omental flaps, which require entering the abdominal cavity but are associated with a higher mortality rate [10]. As a class I and IIb recommendation for deep sternal wound infection, the European Association of Cardiothoracic Surgery recommends NPWT and muscle flap procedures [10]. In instances of sternal dehiscence, the use of muscle flaps avoids the need to dissect adhesions, preventing damage to vital structures and ensuring sternum stability. Further, the dead space between the hemisternum bones is filled with vascularized tissue, which facilitates wound healing. This approach was followed in our case with multistage revision surgeries, including debridement and NPWT, followed by plastic surgery reconstructions of pectoralis major muscle flap closure of an open sternal wound, which had a satisfactory functional outcome.

## Conclusions

The case highlighted the importance of considering fungal pathogens, such as rare species like *Rhizopus*, in the differential diagnosis of sternal osteomyelitis and mediastinitis after cardiac surgery. Timely diagnosis and appropriate treatment, including surgery and targeted antifungal therapy, are crucial for improving patient outcomes and increasing survival rates.
